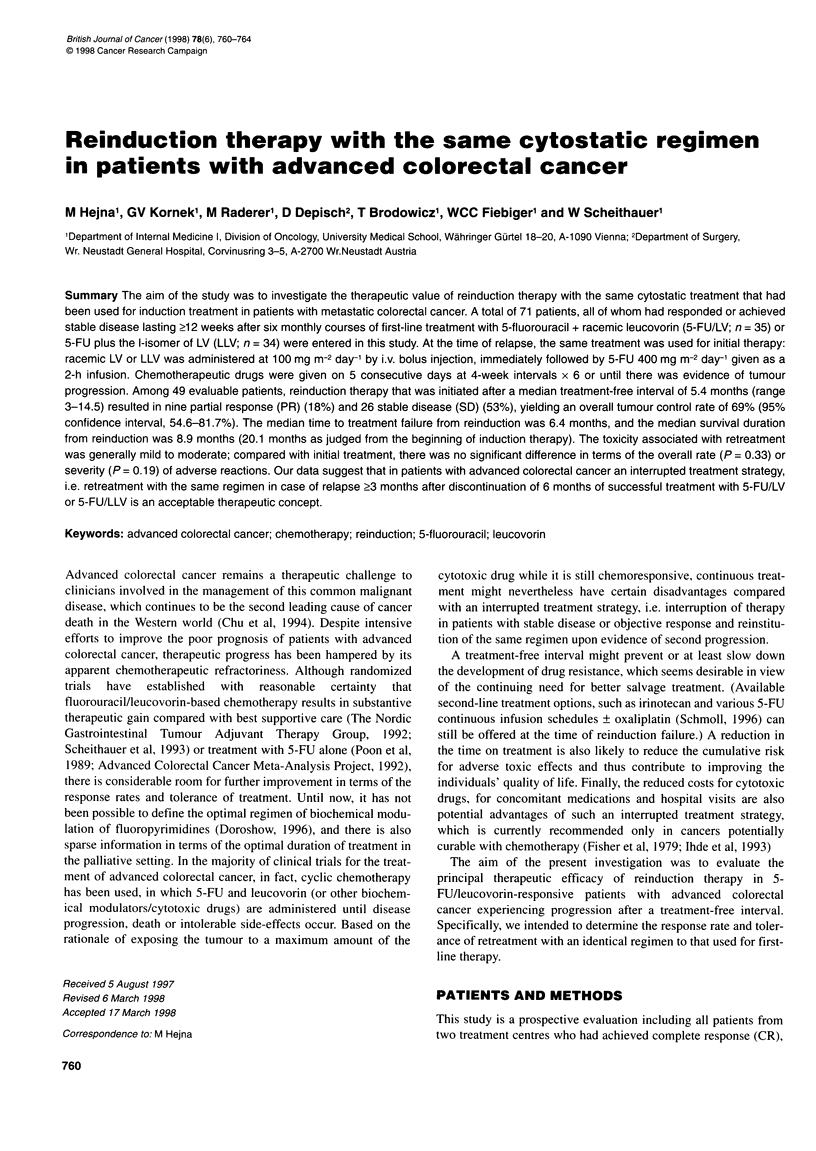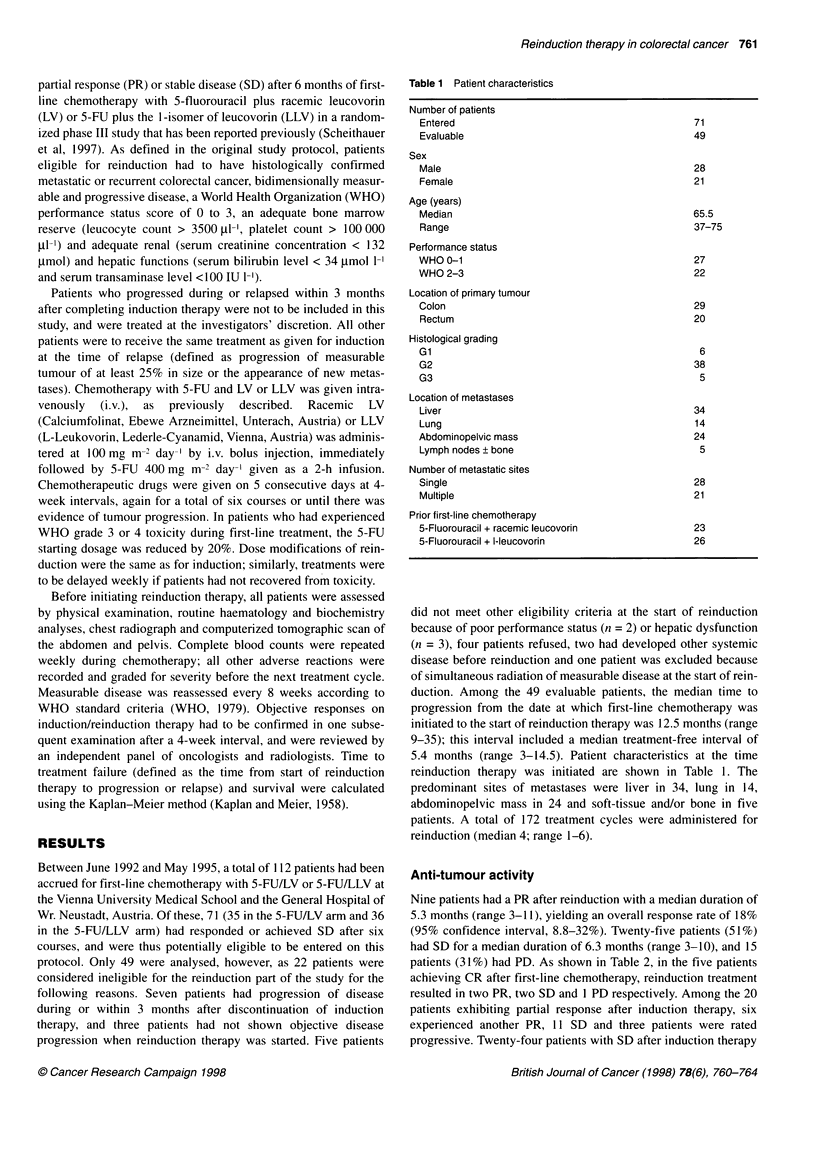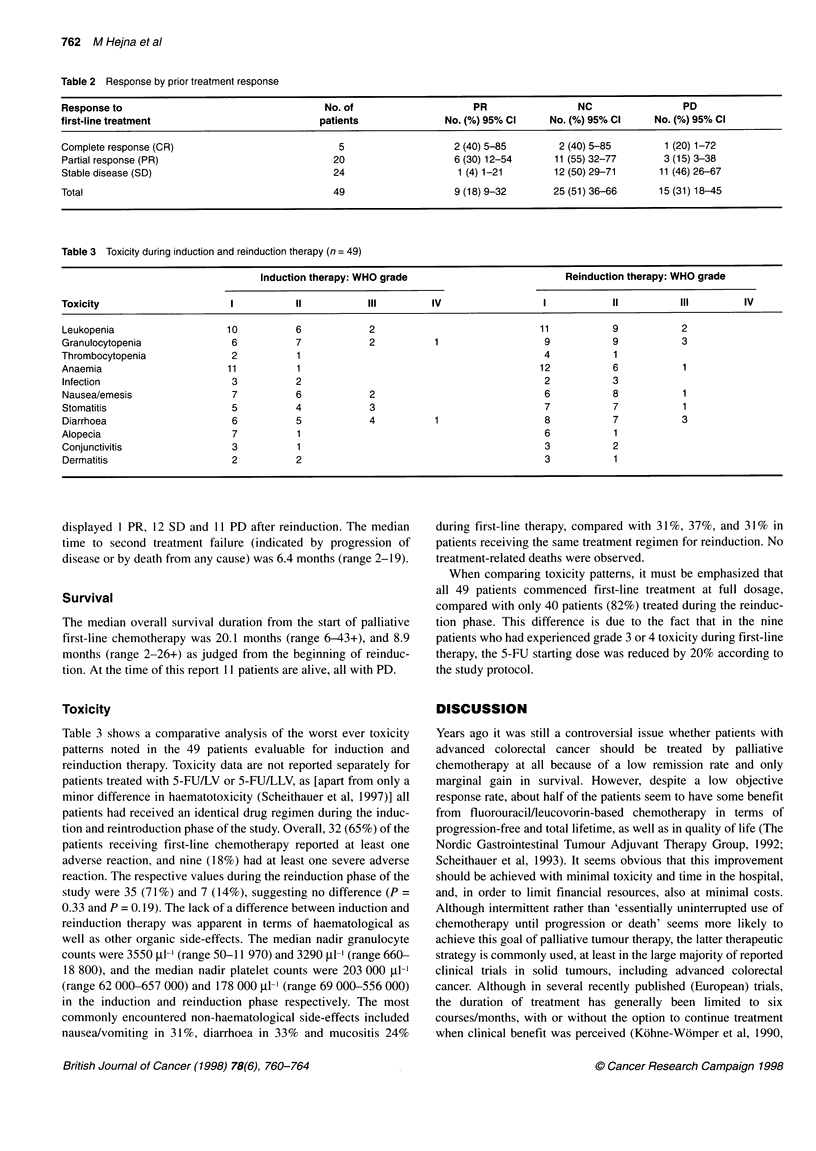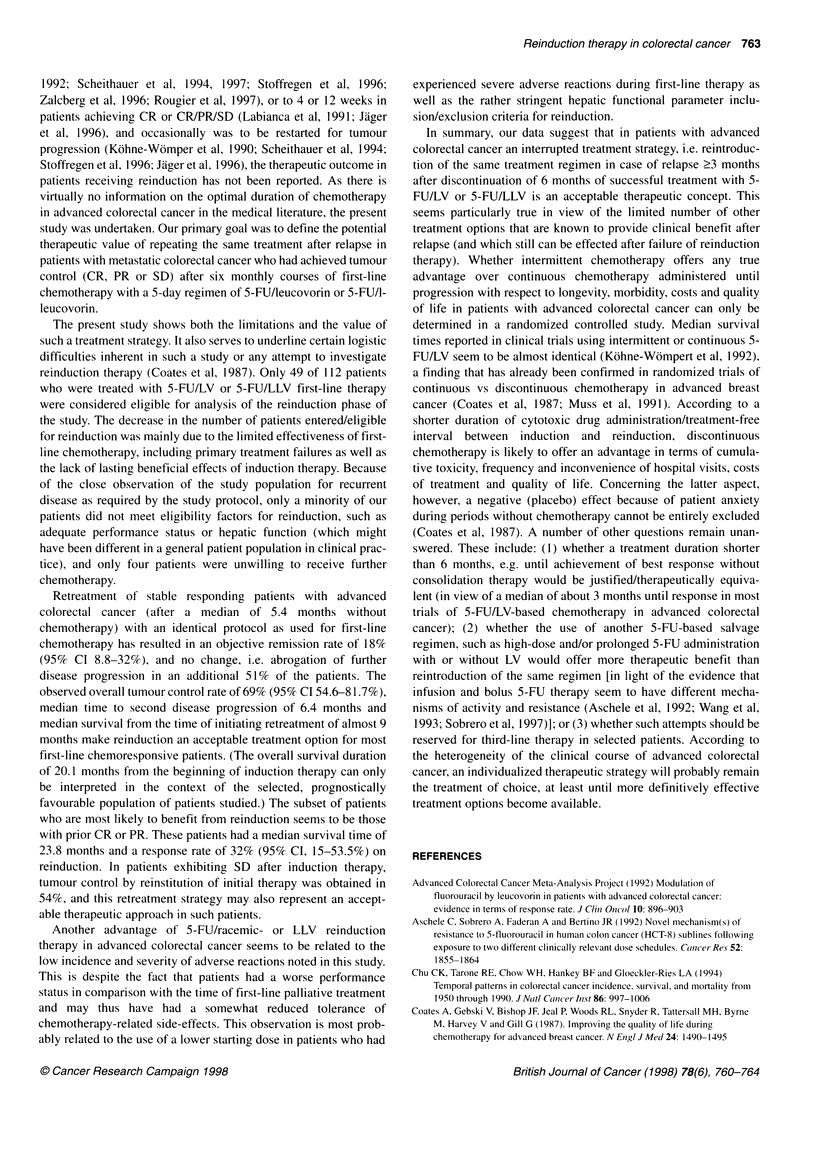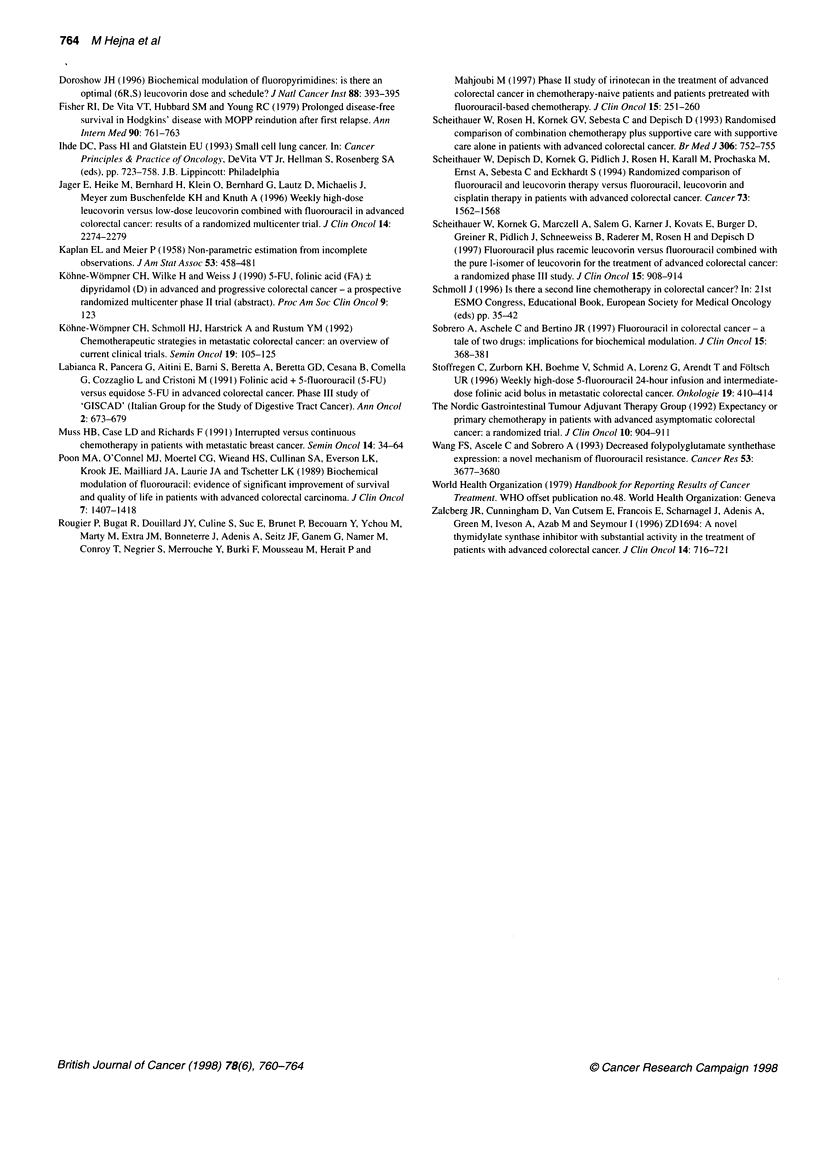# Reinduction therapy with the same cytostatic regimen in patients with advanced colorectal cancer.

**DOI:** 10.1038/bjc.1998.574

**Published:** 1998-09

**Authors:** M. Hejna, G. V. Kornek, M. Raderer, D. Depisch, T. Brodowicz, W. C. Fiebiger, W. Scheithauer

**Affiliations:** Department of Internal Medicine I, University Medical School, Vienna, Austria.

## Abstract

The aim of the study was to investigate the therapeutic value of reinduction therapy with the same cytostatic treatment that had been used for induction treatment in patients with metastatic colorectal cancer. A total of 71 patients, all of whom had responded or achieved stable disease lasting > or = 12 weeks after six monthly courses of first-line treatment with 5-fluorouracil + racemic leucovorin (5-FU/LV; n = 35) or 5-FU plus the l-isomer of LV (LLV; n = 34) were entered in this study. At the time of relapse, the same treatment was used for initial therapy: racemic LV or LLV was administered at 100 mg m(-2) day(-1) by i.v. bolus injection, immediately followed by 5-FU 400 mg m(-2) day(-1) given as a 2-h infusion. Chemotherapeutic drugs were given on 5 consecutive days at 4-week intervals x 6 or until there was evidence of tumour progression. Among 49 evaluable patients, reinduction therapy that was initiated after a median treatment-free interval of 5.4 months (range 3-14.5) resulted in nine partial response (PR) (18%) and 26 stable disease (SD) (53%), yielding an overall tumour control rate of 69% (95% confidence interval, 54.6-81.7%). The median time to treatment failure from reinduction was 6.4 months, and the median survival duration from reinduction was 8.9 months (20.1 months as judged from the beginning of induction therapy). The toxicity associated with retreatment was generally mild to moderate; compared with initial treatment, there was no significant difference in terms of the overall rate (P = 0.33) or severity (P = 0.19) of adverse reactions. Our data suggest that in patients with advanced colorectal cancer an interrupted treatment strategy, i.e. retreatment with the same regimen in case of relapse > or = 3 months after discontinuation of 6 months of successful treatment with 5-FU/LV or 5-FU/LLV is an acceptable therapeutic concept.


British Journal of Cancer (1998) 78(6), 760–764
© 1998 Cancer Research Campaign

### Reinduction therapy with the same cytostatic regimen in patients with advanced colorectal cancer

**M Hejna[1], GV Kornek[1], M Raderer[1], D Depisch[2], T Brodowicz[1], WCC Fiebiger[1] and W Scheithauer[1]**

[1]Department of Internal Medicine I, Division of Oncology, University Medical School, Währinger Gürtel 18–20, A-1090 Vienna; [2]Department of Surgery, Wr. Neustadt General Hospital, Corvinusring 3–5, A-2700 Wr.Neustadt Austria

**Summary** The aim of the study was to investigate the therapeutic value of reinduction therapy with the same cytostatic treatment that had been used for induction treatment in patients with metastatic colorectal cancer. A total of 71 patients, all of whom had responded or achieved stable disease lasting ≥12 weeks after six monthly courses of first-line treatment with 5-fluorouracil + racemic leucovorin (5-FU/LV; $n = 35$) or 5-FU plus the l-isomer of LV (LLV; $n = 34$) were entered in this study. At the time of relapse, the same treatment was used for initial therapy: racemic LV or LLV was administered at 100 mg $m^{-2}$ $day^{-1}$ by i.v. bolus injection, immediately followed by 5-FU 400 mg $m^{-2}$ $day^{-1}$ given as a 2-h infusion. Chemotherapeutic drugs were given on 5 consecutive days at 4-week intervals × 6 or until there was evidence of tumour progression. Among 49 evaluable patients, reinduction therapy that was initiated after a median treatment-free interval of 5.4 months (range 3–14.5) resulted in nine partial response (PR) (18%) and 26 stable disease (SD) (53%), yielding an overall tumour control rate of 69% (95% confidence interval, 54.6–81.7%). The median time to treatment failure from reinduction was 6.4 months, and the median survival duration from reinduction was 8.9 months (20.1 months as judged from the beginning of induction therapy). The toxicity associated with retreatment was generally mild to moderate; compared with initial treatment, there was no significant difference in terms of the overall rate ($P = 0.33$) or severity ($P = 0.19$) of adverse reactions. Our data suggest that in patients with advanced colorectal cancer an interrupted treatment strategy, i.e. retreatment with the same regimen in case of relapse ≥3 months after discontinuation of 6 months of successful treatment with 5-FU/LV or 5-FU/LLV is an acceptable therapeutic concept.

**Keywords:** advanced colorectal cancer; chemotherapy; reinduction; 5-fluorouracil; leucovorin

Advanced colorectal cancer remains a therapeutic challenge to clinicians involved in the management of this common malignant disease, which continues to be the second leading cause of cancer death in the Western world (Chu et al, 1994). Despite intensive efforts to improve the poor prognosis of patients with advanced colorectal cancer, therapeutic progress has been hampered by its apparent chemotherapeutic refractoriness. Although randomized trials have established with reasonable certainty that fluorouracil/leucovorin-based chemotherapy results in substantive therapeutic gain compared with best supportive care (The Nordic Gastrointestinal Tumour Adjuvant Therapy Group, 1992; Scheithauer et al, 1993) or treatment with 5-FU alone (Poon et al, 1989; Advanced Colorectal Cancer Meta-Analysis Project, 1992), there is considerable room for further improvement in terms of the response rates and tolerance of treatment. Until now, it has not been possible to define the optimal regimen of biochemical modulation of fluoropyrimidines (Doroshow, 1996), and there is also sparse information in terms of the optimal duration of treatment in the palliative setting. In the majority of clinical trials for the treatment of advanced colorectal cancer, in fact, cyclic chemotherapy has been used, in which 5-FU and leucovorin (or other biochemical modulators/cytotoxic drugs) are administered until disease progression, death or intolerable side-effects occur. Based on the rationale of exposing the tumour to a maximum amount of the cytotoxic drug while it is still chemoresponsive, continuous treatment might nevertheless have certain disadvantages compared with an interrupted treatment strategy, i.e. interruption of therapy in patients with stable disease or objective response and reinstitution of the same regimen upon evidence of second progression.

A treatment-free interval might prevent or at least slow down the development of drug resistance, which seems desirable in view of the continuing need for better salvage treatment. (Available second-line treatment options, such as irinotecan and various 5-FU continuous infusion schedules ± oxaliplatin (Schmoll, 1996) can still be offered at the time of reinduction failure.) A reduction in the time on treatment is also likely to reduce the cumulative risk for adverse toxic effects and thus contribute to improving the individuals' quality of life. Finally, the reduced costs for cytotoxic drugs, for concomitant medications and hospital visits are also potential advantages of such an interrupted treatment strategy, which is currently recommended only in cancers potentially curable with chemotherapy (Fisher et al, 1979; Ihde et al, 1993)

The aim of the present investigation was to evaluate the principal therapeutic efficacy of reinduction therapy in 5-FU/leucovorin-responsive patients with advanced colorectal cancer experiencing progression after a treatment-free interval. Specifically, we intended to determine the response rate and tolerance of retreatment with an identical regimen to that used for first-line therapy.

*Received 5 August 1997*
*Revised 6 March 1998*
*Accepted 17 March 1998*

*Correspondence to:* M Hejna

### PATIENTS AND METHODS

This study is a prospective evaluation including all patients from two treatment centres who had achieved complete response (CR), partial response (PR) or stable disease (SD) after 6 months of first-line chemotherapy with 5-fluorouracil plus racemic leucovorin (LV) or 5-FU plus the l-isomer of leucovorin (LLV) in a randomized phase III study that has been reported previously (Scheithauer et al, 1997). As defined in the original study protocol, patients eligible for reinduction had to have histologically confirmed metastatic or recurrent colorectal cancer, bidimensionally measurable and progressive disease, a World Health Organization (WHO) performance status score of 0 to 3, an adequate bone marrow reserve (leucocyte count > 3500 $\mu l^{-1}$, platelet count > 100 000 $\mu l^{-1}$) and adequate renal (serum creatinine concentration < 132 μmol) and hepatic functions (serum bilirubin level < 34 μmol $l^{-1}$ and serum transaminase level <100 IU $l^{-1}$).

Patients who progressed during or relapsed within 3 months after completing induction therapy were not to be included in this study, and were treated at the investigators' discretion. All other patients were to receive the same treatment as given for induction at the time of relapse (defined as progression of measurable tumour of at least 25% in size or the appearance of new metastases). Chemotherapy with 5-FU and LV or LLV was given intravenously (i.v.), as previously described. Racemic LV (Calciumfolinat, Ebewe Arzneimittel, Unterach, Austria) or LLV (L-Leukovorin, Lederle-Cyanamid, Vienna, Austria) was administered at 100 mg $m^{-2}$ $day^{-1}$ by i.v. bolus injection, immediately followed by 5-FU 400 mg $m^{-2}$ $day^{-1}$ given as a 2-h infusion. Chemotherapeutic drugs were given on 5 consecutive days at 4-week intervals, again for a total of six courses or until there was evidence of tumour progression. In patients who had experienced WHO grade 3 or 4 toxicity during first-line treatment, the 5-FU starting dosage was reduced by 20%. Dose modifications of reinduction were the same as for induction; similarly, treatments were to be delayed weekly if patients had not recovered from toxicity.

Before initiating reinduction therapy, all patients were assessed by physical examination, routine haematology and biochemistry analyses, chest radiograph and computerized tomographic scan of the abdomen and pelvis. Complete blood counts were repeated weekly during chemotherapy; all other adverse reactions were recorded and graded for severity before the next treatment cycle. Measurable disease was reassessed every 8 weeks according to WHO standard criteria (WHO, 1979). Objective responses on induction/reinduction therapy had to be confirmed in one subsequent examination after a 4-week interval, and were reviewed by an independent panel of oncologists and radiologists. Time to treatment failure (defined as the time from start of reinduction therapy to progression or relapse) and survival were calculated using the Kaplan–Meier method (Kaplan and Meier, 1958).

### RESULTS

Between June 1992 and May 1995, a total of 112 patients had been accrued for first-line chemotherapy with 5-FU/LV or 5-FU/LLV at the Vienna University Medical School and the General Hospital of Wr. Neustadt, Austria. Of these, 71 (35 in the 5-FU/LV arm and 36 in the 5-FU/LLV arm) had responded or achieved SD after six courses, and were thus potentially eligible to be entered on this protocol. Only 49 were analysed, however, as 22 patients were considered ineligible for the reinduction part of the study for the following reasons. Seven patients had progression of disease during or within 3 months after discontinuation of induction therapy, and three patients had not shown objective disease progression when reinduction therapy was started. Five patients did not meet other eligibility criteria at the start of reinduction because of poor performance status ($n$ = 2) or hepatic dysfunction ($n$ = 3), four patients refused, two had developed other systemic disease before reinduction and one patient was excluded because of simultaneous radiation of measurable disease at the start of reinduction. Among the 49 evaluable patients, the median time to progression from the date at which first-line chemotherapy was initiated to the start of reinduction therapy was 12.5 months (range 9–35); this interval included a median treatment-free interval of 5.4 months (range 3–14.5). Patient characteristics at the time reinduction therapy was initiated are shown in Table 1. The predominant sites of metastases were liver in 34, lung in 14, abdominopelvic mass in 24 and soft-tissue and/or bone in five patients. A total of 172 treatment cycles were administered for reinduction (median 4; range 1–6).

**Table 1** Patient characteristics

| | |
|---|---|
| Number of patients | |
| Entered | 71 |
| Evaluable | 49 |
| Sex | |
| Male | 28 |
| Female | 21 |
| Age (years) | |
| Median | 65.5 |
| Range | 37–75 |
| Performance status | |
| WHO 0–1 | 27 |
| WHO 2–3 | 22 |
| Location of primary tumour | |
| Colon | 29 |
| Rectum | 20 |
| Histological grading | |
| G1 | 6 |
| G2 | 38 |
| G3 | 5 |
| Location of metastases | |
| Liver | 34 |
| Lung | 14 |
| Abdominopelvic mass | 24 |
| Lymph nodes ± bone | 5 |
| Number of metastatic sites | |
| Single | 28 |
| Multiple | 21 |
| Prior first-line chemotherapy | |
| 5-Fluorouracil + racemic leucovorin | 23 |
| 5-Fluorouracil + l-leucovorin | 26 |

### Anti-tumour activity

Nine patients had a PR after reinduction with a median duration of 5.3 months (range 3–11), yielding an overall response rate of 18% (95% confidence interval, 8.8–32%). Twenty-five patients (51%) had SD for a median duration of 6.3 months (range 3–10), and 15 patients (31%) had PD. As shown in Table 2, in the five patients achieving CR after first-line chemotherapy, reinduction treatment resulted in two PR, two SD and 1 PD respectively. Among the 20 patients exhibiting partial response after induction therapy, six experienced another PR, 11 SD and three patients were rated progressive. Twenty-four patients with SD after induction therapy

© Cancer Research Campaign 1998

Table 2 Response by prior treatment response

| Response to first-line treatment | No. of patients | PR No. (%) 95% CI | NC No. (%) 95% CI | PD No. (%) 95% CI |
|---|---|---|---|---|
| Complete response (CR) | 5 | 2 (40) 5–85 | 2 (40) 5–85 | 1 (20) 1–72 |
| Partial response (PR) | 20 | 6 (30) 12–54 | 11 (55) 32–77 | 3 (15) 3–38 |
| Stable disease (SD) | 24 | 1 (4) 1–21 | 12 (50) 29–71 | 11 (46) 26–67 |
| Total | 49 | 9 (18) 9–32 | 25 (51) 36–66 | 15 (31) 18–45 |

Table 3 Toxicity during induction and reinduction therapy ($n$ = 49)

| | Induction therapy: WHO grade | | | | Reinduction therapy: WHO grade | | | |
|---|---|---|---|---|---|---|---|---|
| Toxicity | I | II | III | IV | I | II | III | IV |
| Leukopenia | 10 | 6 | 2 | | 11 | 9 | 2 | |
| Granulocytopenia | 6 | 7 | 2 | 1 | 9 | 9 | 3 | |
| Thrombocytopenia | 2 | 1 | | | 4 | 1 | | |
| Anaemia | 11 | 1 | | | 12 | 6 | 1 | |
| Infection | 3 | 2 | | | 2 | 3 | | |
| Nausea/emesis | 7 | 6 | 2 | | 6 | 8 | 1 | |
| Stomatitis | 5 | 4 | 3 | | 7 | 7 | 1 | |
| Diarrhoea | 6 | 5 | 4 | 1 | 8 | 7 | 3 | |
| Alopecia | 7 | 1 | | | 6 | 1 | | |
| Conjunctivitis | 3 | 1 | | | 3 | 2 | | |
| Dermatitis | 2 | 2 | | | 3 | 1 | | |

displayed 1 PR, 12 SD and 11 PD after reinduction. The median time to second treatment failure (indicated by progression of disease or by death from any cause) was 6.4 months (range 2–19).

### Survival

The median overall survival duration from the start of palliative first-line chemotherapy was 20.1 months (range 6–43+), and 8.9 months (range 2–26+) as judged from the beginning of reinduction. At the time of this report 11 patients are alive, all with PD.

### Toxicity

Table 3 shows a comparative analysis of the worst ever toxicity patterns noted in the 49 patients evaluable for induction and reinduction therapy. Toxicity data are not reported separately for patients treated with 5-FU/LV or 5-FU/LLV, as [apart from only a minor difference in haematotoxicity (Scheithauer et al, 1997)] all patients had received an identical drug regimen during the induction and reintroduction phase of the study. Overall, 32 (65%) of the patients receiving first-line chemotherapy reported at least one adverse reaction, and nine (18%) had at least one severe adverse reaction. The respective values during the reinduction phase of the study were 35 (71%) and 7 (14%), suggesting no difference ($P$ = 0.33 and $P$ = 0.19). The lack of a difference between induction and reinduction therapy was apparent in terms of haematological as well as other organic side-effects. The median nadir granulocyte counts were 3550 $\mu l^{-1}$ (range 50–11 970) and 3290 $\mu l^{-1}$ (range 660–18 800), and the median nadir platelet counts were 203 000 $\mu l^{-1}$ (range 62 000–657 000) and 178 000 $\mu l^{-1}$ (range 69 000–556 000) in the induction and reinduction phase respectively. The most commonly encountered non-haematological side-effects included nausea/vomiting in 31%, diarrhoea in 33% and mucositis 24% during first-line therapy, compared with 31%, 37%, and 31% in patients receiving the same treatment regimen for reinduction. No treatment-related deaths were observed.

When comparing toxicity patterns, it must be emphasized that all 49 patients commenced first-line treatment at full dosage, compared with only 40 patients (82%) treated during the reinduction phase. This difference is due to the fact that in the nine patients who had experienced grade 3 or 4 toxicity during first-line therapy, the 5-FU starting dose was reduced by 20% according to the study protocol.

### DISCUSSION

Years ago it was still a controversial issue whether patients with advanced colorectal cancer should be treated by palliative chemotherapy at all because of a low remission rate and only marginal gain in survival. However, despite a low objective response rate, about half of the patients seem to have some benefit from fluorouracil/leucovorin-based chemotherapy in terms of progression-free and total lifetime, as well as in quality of life (The Nordic Gastrointestinal Tumour Adjuvant Therapy Group, 1992; Scheithauer et al, 1993). It seems obvious that this improvement should be achieved with minimal toxicity and time in the hospital, and, in order to limit financial resources, also at minimal costs. Although intermittent rather than 'essentially uninterrupted use of chemotherapy until progression or death' seems more likely to achieve this goal of palliative tumour therapy, the latter therapeutic strategy is commonly used, at least in the large majority of reported clinical trials in solid tumours, including advanced colorectal cancer. Although in several recently published (European) trials, the duration of treatment has generally been limited to six courses/months, with or without the option to continue treatment when clinical benefit was perceived (Köhne-Wömper et al, 1990,

© Cancer Research Campaign 1998

1992; Scheithauer et al, 1994, 1997; Stoffregen et al, 1996; Zalcberg et al, 1996; Rougier et al, 1997), or to 4 or 12 weeks in patients achieving CR or CR/PR/SD (Labianca et al, 1991; Jäger et al, 1996), and occasionally was to be restarted for tumour progression (Köhne-Wömper et al, 1990; Scheithauer et al, 1994; Stoffregen et al, 1996; Jäger et al, 1996), the therapeutic outcome in patients receiving reinduction has not been reported. As there is virtually no information on the optimal duration of chemotherapy in advanced colorectal cancer in the medical literature, the present study was undertaken. Our primary goal was to define the potential therapeutic value of repeating the same treatment after relapse in patients with metastatic colorectal cancer who had achieved tumour control (CR, PR or SD) after six monthly courses of first-line chemotherapy with a 5-day regimen of 5-FU/leucovorin or 5-FU/l-leucovorin.

The present study shows both the limitations and the value of such a treatment strategy. It also serves to underline certain logistic difficulties inherent in such a study or any attempt to investigate reinduction therapy (Coates et al, 1987). Only 49 of 112 patients who were treated with 5-FU/LV or 5-FU/LLV first-line therapy were considered eligible for analysis of the reinduction phase of the study. The decrease in the number of patients entered/eligible for reinduction was mainly due to the limited effectiveness of first-line chemotherapy, including primary treatment failures as well as the lack of lasting beneficial effects of induction therapy. Because of the close observation of the study population for recurrent disease as required by the study protocol, only a minority of our patients did not meet eligibility factors for reinduction, such as adequate performance status or hepatic function (which might have been different in a general patient population in clinical practice), and only four patients were unwilling to receive further chemotherapy.

Retreatment of stable responding patients with advanced colorectal cancer (after a median of 5.4 months without chemotherapy) with an identical protocol as used for first-line chemotherapy has resulted in an objective remission rate of 18% (95% CI 8.8–32%), and no change, i.e. abrogation of further disease progression in an additional 51% of the patients. The observed overall tumour control rate of 69% (95% CI 54.6–81.7%), median time to second disease progression of 6.4 months and median survival from the time of initiating retreatment of almost 9 months make reinduction an acceptable treatment option for most first-line chemoresponsive patients. (The overall survival duration of 20.1 months from the beginning of induction therapy can only be interpreted in the context of the selected, prognostically favourable population of patients studied.) The subset of patients who are most likely to benefit from reinduction seems to be those with prior CR or PR. These patients had a median survival time of 23.8 months and a response rate of 32% (95% CI, 15–53.5%) on reinduction. In patients exhibiting SD after induction therapy, tumour control by reinstitution of initial therapy was obtained in 54%, and this retreatment strategy may also represent an acceptable therapeutic approach in such patients.

Another advantage of 5-FU/racemic- or LLV reinduction therapy in advanced colorectal cancer seems to be related to the low incidence and severity of adverse reactions noted in this study. This is despite the fact that patients had a worse performance status in comparison with the time of first-line palliative treatment and may thus have had a somewhat reduced tolerance of chemotherapy-related side-effects. This observation is most probably related to the use of a lower starting dose in patients who had experienced severe adverse reactions during first-line therapy as well as the rather stringent hepatic functional parameter inclusion/exclusion criteria for reinduction.

In summary, our data suggest that in patients with advanced colorectal cancer an interrupted treatment strategy, i.e. reintroduction of the same treatment regimen in case of relapse ≥3 months after discontinuation of 6 months of successful treatment with 5-FU/LV or 5-FU/LLV is an acceptable therapeutic concept. This seems particularly true in view of the limited number of other treatment options that are known to provide clinical benefit after relapse (and which still can be effected after failure of reinduction therapy). Whether intermittent chemotherapy offers any true advantage over continuous chemotherapy administered until progression with respect to longevity, morbidity, costs and quality of life in patients with advanced colorectal cancer can only be determined in a randomized controlled study. Median survival times reported in clinical trials using intermittent or continuous 5-FU/LV seem to be almost identical (Köhne-Wömpert et al, 1992), a finding that has already been confirmed in randomized trials of continuous vs discontinuous chemotherapy in advanced breast cancer (Coates et al, 1987; Muss et al, 1991). According to a shorter duration of cytotoxic drug administration/treatment-free interval between induction and reinduction, discontinuous chemotherapy is likely to offer an advantage in terms of cumulative toxicity, frequency and inconvenience of hospital visits, costs of treatment and quality of life. Concerning the latter aspect, however, a negative (placebo) effect because of patient anxiety during periods without chemotherapy cannot be entirely excluded (Coates et al, 1987). A number of other questions remain unanswered. These include: (1) whether a treatment duration shorter than 6 months, e.g. until achievement of best response without consolidation therapy would be justified/therapeutically equivalent (in view of a median of about 3 months until response in most trials of 5-FU/LV-based chemotherapy in advanced colorectal cancer); (2) whether the use of another 5-FU-based salvage regimen, such as high-dose and/or prolonged 5-FU administration with or without LV would offer more therapeutic benefit than reintroduction of the same regimen [in light of the evidence that infusion and bolus 5-FU therapy seem to have different mechanisms of activity and resistance (Aschele et al, 1992; Wang et al, 1993; Sobrero et al, 1997)]; or (3) whether such attempts should be reserved for third-line therapy in selected patients. According to the heterogeneity of the clinical course of advanced colorectal cancer, an individualized therapeutic strategy will probably remain the treatment of choice, at least until more definitively effective treatment options become available.

### REFERENCES

Advanced Colorectal Cancer Meta-Analysis Project (1992) Modulation of fluorouracil by leucovorin in patients with advanced colorectal cancer: evidence in terms of response rate. *J Clin Oncol* **10**: 896–903

Aschele C, Sobrero A, Faderan A and Bertino JR (1992) Novel mechanism(s) of resistance to 5-fluorouracil in human colon cancer (HCT-8) sublines following exposure to two different clinically relevant dose schedules. *Cancer Res* **52**: 1855–1864

Chu CK, Tarone RE, Chow WH, Hankey BF and Gloeckler-Ries LA (1994) Temporal patterns in colorectal cancer incidence, survival, and mortality from 1950 through 1990. *J Natl Cancer Inst* **86**: 997–1006

Coates A, Gebski V, Bishop JF, Jeal P, Woods RL, Snyder R, Tattersall MH, Byrne M, Harvey V and Gill G (1987). Improving the quality of life during chemotherapy for advanced breast cancer. *N Engl J Med* **24**: 1490–1495

Doroshow JH (1996) Biochemical modulation of fluoropyrimidines: is there an optimal (6R,S) leucovorin dose and schedule? *J Natl Cancer Inst* **88**: 393–395

Fisher RI, De Vita VT, Hubbard SM and Young RC (1979) Prolonged disease-free survival in Hodgkins' disease with MOPP reindution after first relapse. *Ann Intern Med* **90**: 761–763

Ihde DC, Pass HI and Glatstein EU (1993) Small cell lung cancer. In: *Cancer Principles & Practice of Oncology*, DeVita VT Jr, Hellman S, Rosenberg SA (eds), pp. 723–758. J.B. Lippincott: Philadelphia

Jager E, Heike M, Bernhard H, Klein O, Bernhard G, Lautz D, Michaelis J, Meyer zum Buschenfelde KH and Knuth A (1996) Weekly high-dose leucovorin versus low-dose leucovorin combined with fluorouracil in advanced colorectal cancer: results of a randomized multicenter trial. *J Clin Oncol* **14**: 2274–2279

Kaplan EL and Meier P (1958) Non-parametric estimation from incomplete observations. *J Am Stat Assoc* **53**: 458–481

Köhne-Wömpner CH, Wilke H and Weiss J (1990) 5-FU, folinic acid (FA) ± dipyridamol (D) in advanced and progressive colorectal cancer – a prospective randomized multicenter phase II trial (abstract). *Proc Am Soc Clin Oncol* **9**: 123

Köhne-Wömpner CH, Schmoll HJ, Harstrick A and Rustum YM (1992) Chemotherapeutic strategies in metastatic colorectal cancer: an overview of current clinical trials. *Semin Oncol* **19**: 105–125

Labianca R, Pancera G, Aitini E, Barni S, Beretta A, Beretta GD, Cesana B, Comella G, Cozzaglio L and Cristoni M (1991) Folinic acid + 5-fluorouracil (5-FU) versus equidose 5-FU in advanced colorectal cancer. Phase III study of 'GISCAD' (Italian Group for the Study of Digestive Tract Cancer). *Ann Oncol* **2**: 673–679

Muss HB, Case LD and Richards F (1991) Interrupted versus continuous chemotherapy in patients with metastatic breast cancer. *Semin Oncol* **14**: 34–64

Poon MA, O'Connel MJ, Moertel CG, Wieand HS, Cullinan SA, Everson LK, Krook JE, Mailliard JA, Laurie JA and Tschetter LK (1989) Biochemical modulation of fluorouracil: evidence of significant improvement of survival and quality of life in patients with advanced colorectal carcinoma. *J Clin Oncol* **7**: 1407–1418

Rougier P, Bugat R, Douillard JY, Culine S, Suc E, Brunet P, Becouarn Y, Ychou M, Marty M, Extra JM, Bonneterre J, Adenis A, Seitz JF, Ganem G, Namer M, Conroy T, Negrier S, Merrouche Y, Burki F, Mousseau M, Herait P and Mahjoubi M (1997) Phase II study of irinotecan in the treatment of advanced colorectal cancer in chemotherapy-naive patients and patients pretreated with fluorouracil-based chemotherapy. *J Clin Oncol* **15**: 251–260

Scheithauer W, Rosen H, Kornek GV, Sebesta C and Depisch D (1993) Randomised comparison of combination chemotherapy plus supportive care with supportive care alone in patients with advanced colorectal cancer. *Br Med J* **306**: 752–755

Scheithauer W, Depisch D, Kornek G, Pidlich J, Rosen H, Karall M, Prochaska M, Ernst A, Sebesta C and Eckhardt S (1994) Randomized comparison of fluorouracil and leucovorin therapy versus fluorouracil, leucovorin and cisplatin therapy in patients with advanced colorectal cancer. *Cancer* **73**: 1562–1568

Scheithauer W, Kornek G, Marczell A, Salem G, Karner J, Kovats E, Burger D, Greiner R, Pidlich J, Schneeweiss B, Raderer M, Rosen H and Depisch D (1997) Fluorouracil plus racemic leucovorin versus fluorouracil combined with the pure l-isomer of leucovorin for the treatment of advanced colorectal cancer: a randomized phase III study. *J Clin Oncol* **15**: 908–914

Schmoll J (1996) Is there a second line chemotherapy in colorectal cancer? In: 21st ESMO Congress, Educational Book, European Society for Medical Oncology (eds) pp. 35–42

Sobrero A, Aschele C and Bertino JR (1997) Fluorouracil in colorectal cancer – a tale of two drugs: implications for biochemical modulation. *J Clin Oncol* **15**: 368–381

Stoffregen C, Zurborn KH, Boehme V, Schmid A, Lorenz G, Arendt T and Föltsch UR (1996) Weekly high-dose 5-fluorouracil 24-hour infusion and intermediate-dose folinic acid bolus in metastatic colorectal cancer. *Onkologie* **19**: 410–414

The Nordic Gastrointestinal Tumour Adjuvant Therapy Group (1992) Expectancy or primary chemotherapy in patients with advanced asymptomatic colorectal cancer: a randomized trial. *J Clin Oncol* **10**: 904–911

Wang FS, Ascele C and Sobrero A (1993) Decreased folypolyglutamate synthethase expression: a novel mechanism of fluorouracil resistance. *Cancer Res* **53**: 3677–3680

World Health Organization (1979) *Handbook for Reporting Results of Cancer Treatment.* WHO offset publication no.48. World Health Organization: Geneva

Zalcberg JR, Cunningham D, Van Cutsem E, Francois E, Scharnagel J, Adenis A, Green M, Iveson A, Azab M and Seymour I (1996) ZD1694: A novel thymidylate synthase inhibitor with substantial activity in the treatment of patients with advanced colorectal cancer. *J Clin Oncol* **14**: 716–721

© Cancer Research Campaign 1998